# Supplementary figures and images for: Digital process control of multi-step assays on centrifugal platforms using high-low-high rotational-pulse triggered valving
Source: PLoS One. 2023 Sep 8;18(9):e0291165. doi: 10.1371/journal.pone.0291165 (PMC10490917; doi:10.1371/journal.pone.0291165)

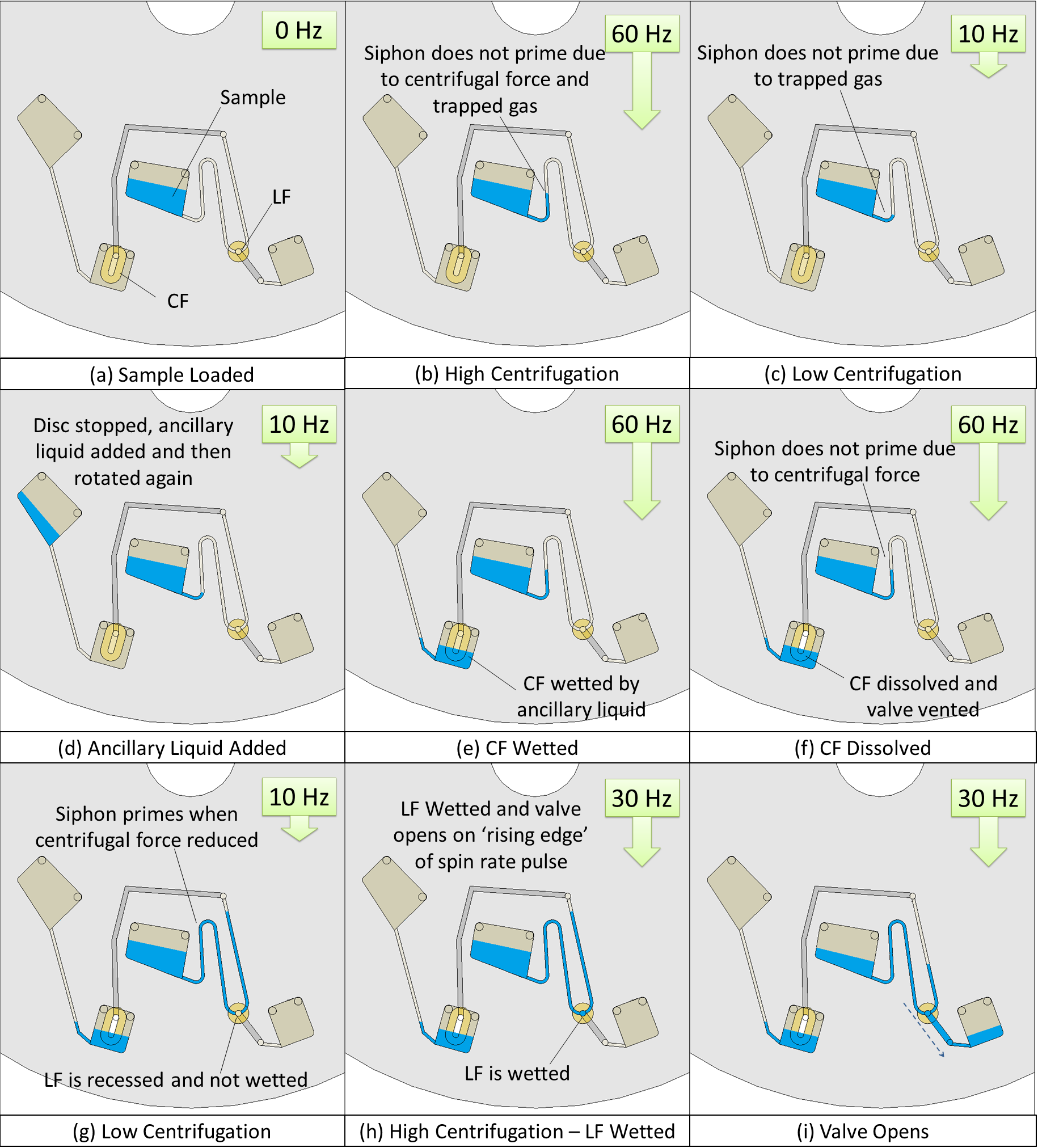

Supplement: S1 Fig — Here, with the valve pneumatically sealed by the CF valve, the siphon will not prime at low or high centrifugation rates. When the CF is wetted and dissolved, the valve is vented to atmosphere and so the siphon will prime at low disc spin rates. An advantage is that, due to the manufacturing process, the DF is recessed from contact with the liquid. Therefore, the LF is wetted on the ‘rising edge’ of the downward pulse in spin rate. This characteristic increases the temporal accuracy of the valves. (TIF) [file pone.0291165.s001.tif]

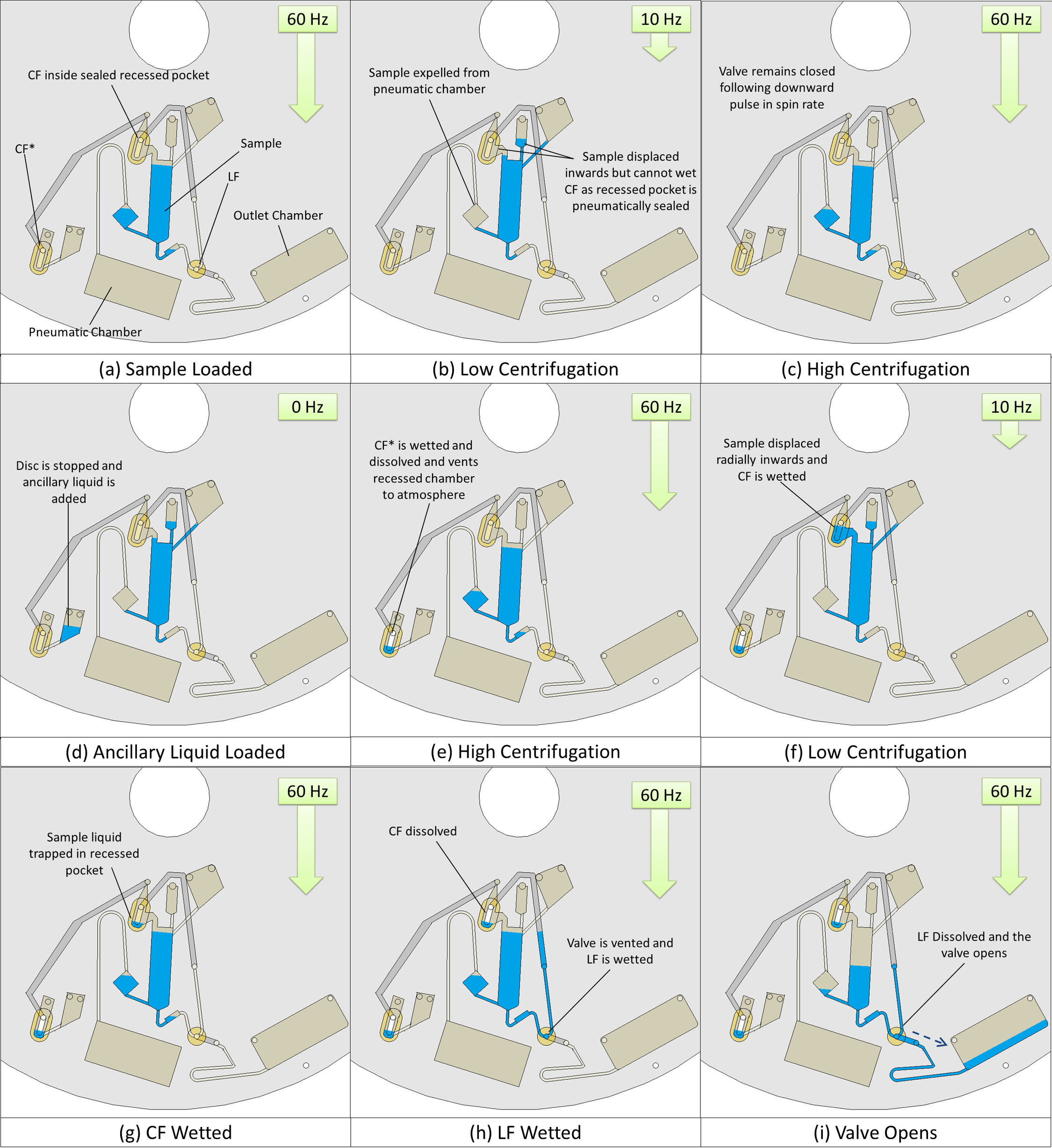

Supplement: S2 Fig — Here, the core of the valve is a conventional event-triggered valve. This is composed of a Load Film (LF) and a Control Film (CF). The valves functions as the liquid height in the reservoir is controlled by a pneumatic chamber. The CF is recessed in a dead-end pneumatic chamber so that liquid cannot reach it. This pneumatic chamber is sealed by a third dissolvable film, called CF*. At high spin rates, the liquid level is below the CF. At low spin-rates, the liquid can be displaced inwards but cannot wet the CF due to the trapped gas in the recessed pneumatic chamber. However, with CF* wetted and dissolved, the CF can be wetted and so the valve can be opened. (TIF) [file pone.0291165.s002.tif]

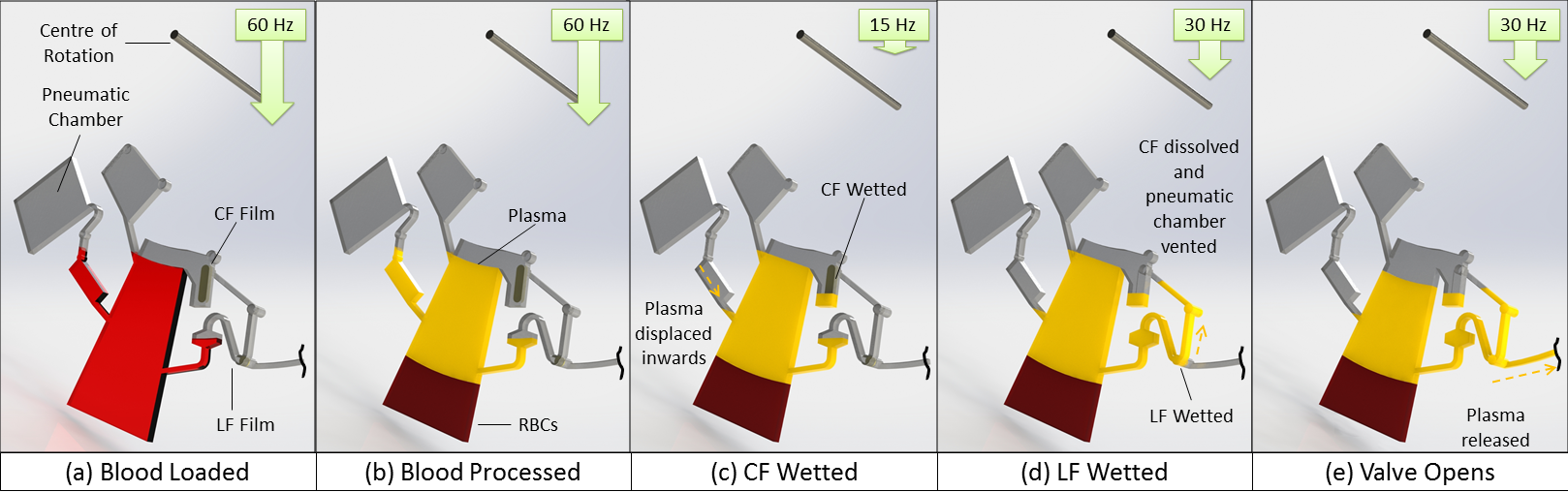

Supplement: S3 Fig — Blood is loaded at a high spin rate (60 Hz). A portion of blood is compressed into a dead end pneumatic chamber. Following separation the spin rate is reduced. This liquid in the pneumatic chamber, now primarily composed of plasm), is pumped back into the main chamber and the liquid height increases. The overflow of plasma wets the CF film. When this dissolves, the valve is vented and the plasma is released form the blood processing structure. This valve is an implementation of the DF-derived low pass pulse actuated valves. (TIF) [file pone.0291165.s003.tif]

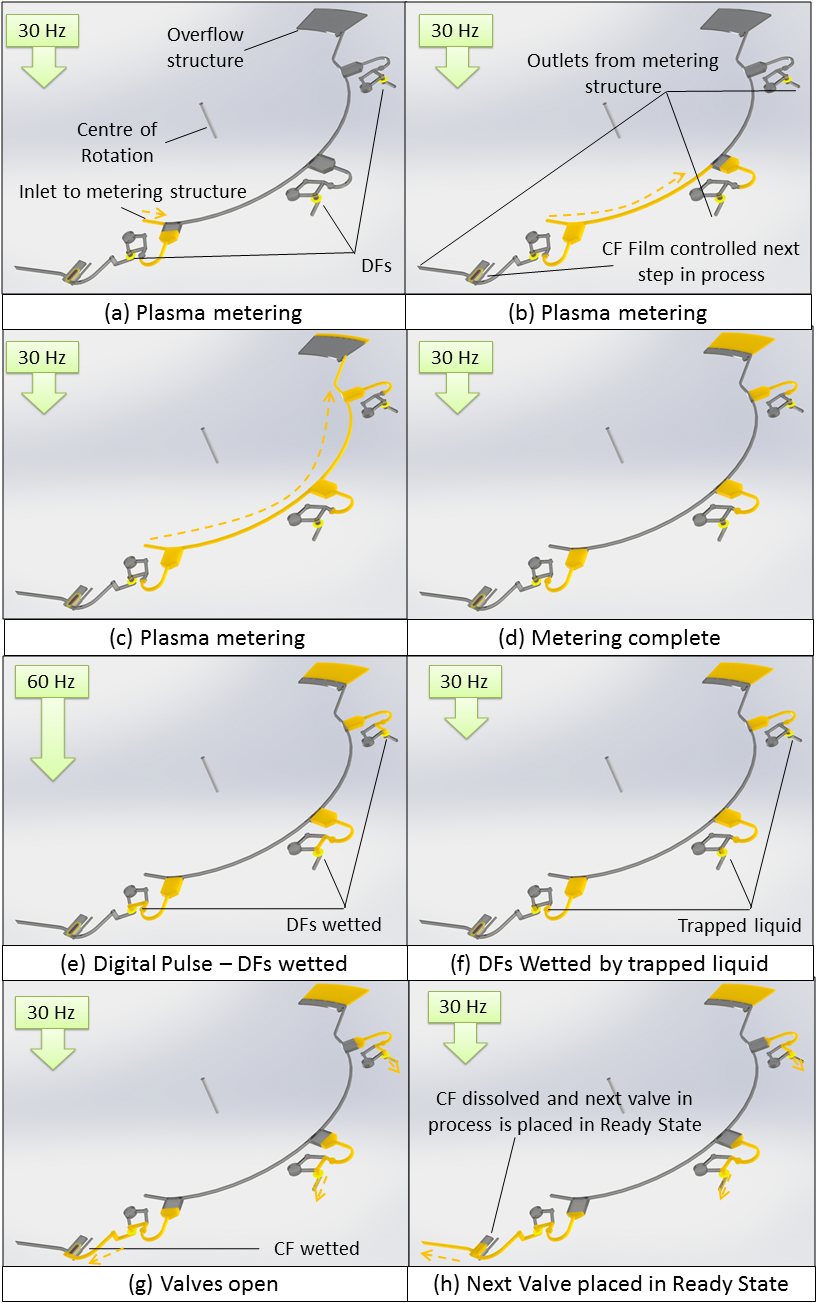

Supplement: S4 Fig — Following metering, an upward digital pulse in the spin rate pushes liquid into the dead end pneumatic chambers (DF Valves) where plasma wets the LFs. The chamber is designed so that, on a reduction of the spin rate, the liquid is trapped in contact with the DF. This improves valve reliability when opened via pulses in spin rate. Additionally, the outgoing plasma wets and dissolves the CF which controls the next process valve. Thus, the next valve is placed in a ‘Ready State’ and can be opened by a pulse in spin rate. This valve is an implementation of DF burst valves described previously in literature. (TIF) [file pone.0291165.s004.tif]

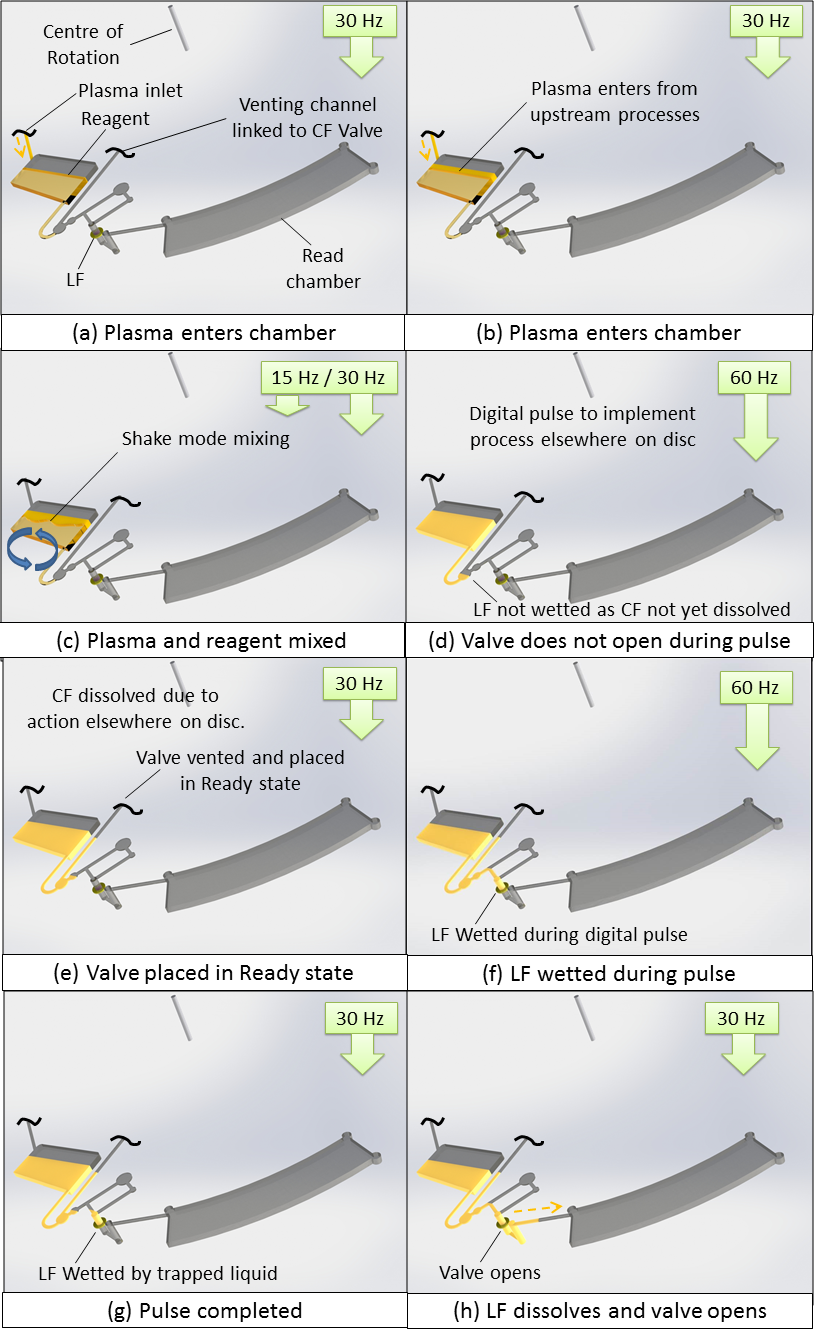

Supplement: S5 Fig — Mixing of plasma with reagent can be improved by rapid ‘shake mode’ mixing where the spin rate is robustly increased and decreased. In this schematic, describing the last valve opened in the sequence, when a digital pulse occurs where the valve CF is not dissolved (panel (d)), the valve does not open. However, with this CF dissolved and vented, which occurs as a result of this digital pulse, the valve is in a ‘Read State’ and will open on the next pulse (panels (f-h). This valve is an implementation of the high pass valves described above. (TIF) [file pone.0291165.s005.tif]
