# Supplementary material for: Digital process control of multi-step assays on centrifugal platforms using high-low-high rotational-pulse triggered valving
Source: PLoS One. 2023 Sep 8;18(9):e0291165. doi: 10.1371/journal.pone.0291165 (PMC10490917; doi:10.1371/journal.pone.0291165)
Supplement: Attachment — Submitted filename: PLOS_Comments_to_reviewers.docx [file pone.0291165.s009.docx]

**Review Comments to the Author**

Reviewer #1: This manuscript mainly demonstrated two different HLH valves designs (Siphon Derived HLH Valve and DF Derived HLH Valves). Controlling by digital pulsing, these valves could achieve a more robust flow control with higher sequential steps. Furthermore, authors integrate the HLH valve with previously developed LHL valves and standard DF burst valves and deploy it to measure DB, TB and ALP based on a liver assay protocol. Here are some technical comments for this manuscript:

1. Authors introduce two different HLH valves: Siphon Derived and DF Derived HLH Valves. It seems that siphon derived valve is highly dependent on the hydrophilicity of the materials, which could has a limited use compared with DF derived one. In the liver assay demonstration, authors didn't use siphon derived HLH valves either. Then it is confusing to show the siphon derived HLH valve in this manuscript unless authors could use experiments to justify its clear advantages or unique properties.

*We thank the reviewer for their comment. We agree that, for this particular application, the DF-derived HLH valves were superior. However, we believe that the siphon-derived valves have relative advantages which are not present in this application but may be present in other applications. We therefore feel it makes sense to present them as part of the same body of work as the HLH DF-derived valves in order to offer the reader a full suite of options. We believe these valves (both types) are the primary innovation focus of this work.*

*Unfortunately, this work was completed as part of a project which is no longer active and so we do not have the capacity to demonstrate the HLH siphon-derived vales as part of an experimental assay. However, to strengthen the case for their inclusion, we have outlined clearly in the conclusions their relative advantages / disadvantages. For convenience, this text is shown here.*

2. In the main figure, authors only show the operation process of HLH valves. However, illustration of he detailed mechanism is missing. Please include more zoom-in schematics to describe what is happening at the place of interest.

*We refer the reviewer to the ESI file in the original submission. These contained panel-by-panel schematics showing simplified versions of the valve operation. We placed these in the ESI as we believed they would be unwieldly in the main text. However, on the recommendation of the editor, we will be pleased to move some or all these schematics into the main text.*

*We note Reviewer #2 also commented the ESI file was missing. We will ensure it is attached to the primary document for consideration in this case.*

3. The photos quality is poor in terms of color contrast and brightness. It is difficult for readers to see the colors and details.

*Unfortunately, this poor image quality is a result of acquiring images using a stroboscopic imaging system acquiring images as the discs spin at up to 3600 rpm. The brightness is a combination of strobe pulse width, camera chip exposure time, camera shutter setting, and the speeds at which the disc is rotating. Often times minor changes, to try and improve the quality of the images, can result in the disc appearing blurred / smeared or, alternatively, the strobe brightness washing out the features. We don't think we can improve on these images based on our existing hardware. We would state however that these images are similar in quality to those published by other groups working in 'Lab-on-a-Disc'. It is difficult to achieve better performance without significant capital investments beyond our current system (which cost ~€8000 to assemble).*

*We also highlight that the images seen by the reviewer were embedded in the uploaded word document and converted to pdf. In the resubmission, we upload the original .png / .jpg files which can be viewed separately (and will be embedded in the html online document as well as the version of record pdf). Figure 2 is ~ 1800 x 900px and Figure 3 is ~2700 x 900px. This resolution allows the reader to zoom in to see most details on the discs clearly. We apologise to the reviewer that we did not upload these files as part of our original submission.*

Reviewer #2: In this manuscript, Early et al. introduced a fabrication technique to use dissolvable membrane to fabricate pulse-actuated valves for liquid processing and handling. Overall, the manuscript is well organized, but there are still some issues to address before it can be accepted.

*We thank the reviewer for their kind comments.*

1. In the manuscript the author used calibrants for the assay demonstration, and it is unclear how close it is related to biofluids such as blood. Therefore, how would the device perform if blood was processed in the device?

*The reviewer is correct that this manuscript reported on processing calibrant only. We conducted this work in ~2014 and had delays in publishing it. We did test this system with blood but cannot report this data as we cannot locate supporting documentation. For example, the blood tested was from an anonymous donor and was tested clinically against the markers in this study before use (both to benchmark this platform and to ensure the samples were within the clinically healthy range). Unfortunately, these results were lost when a member of our research team left the university a number of years ago and their cloud storage was deleted 3 months later (per standard practise / data protection rules in our university). We apologise that we cannot address this drawback. The only evidence of blood-testing is a video of one of our tests (see screenshot). We can state that in our tests, we have identified that blood samples appear to be effected by the stroboscopic light source as previously hypothesized by Kilcawley et al.* [1]*. Note however that Nwankire et al.* [2] *reported excellent data from a similar bio-assay where measurements were made on a portable instrument (with no stroboscopic light source).*

*We have added the following text to the conclusions to highlight that this assay has previously been demonstrated from whole blood:*

2. It is unclear about how the properties of the biofluid would influence the processing results. For example, how would the viscosity affect the results. The author should include a detailed investigation targeting different kinds of biofluids.

*In view of the reviewer’s comment #1 referring explicitly to blood, we assume this comment is referring to biofluid processing generally on the Lab-on-a-Disc and specifically in these HLH valves.*

*Generally speaking, in Lab-on-a-disc, the viscosity and density of a given liquid have little or not impact. The contact angle will have an impact of the priming of siphon valves at low spin-rates but otherwise has no effects. The reason for this is the centrifugal force which acts to pump the liquid acts will overcome any viscous effects.*

*Typical water-based biological reagents will have a viscosity of ~1 mPa****/****s while plasma is ~1.33 mPa/s at the top of the healthy range. Of liquids which might be tested at point-of-care / point-of-use, milk is likely the most viscous at ~2.12 mPa/s.*

*At typical disc spin rates, and using channels of the dimensions in this work (0.5mm x 0.086mm), the time for a 10 µl volume of water to flow from a chamber at the 30mm radius to the 50mm radius along the shortest distance possible, with both chambers fully vented, is likely 2-3 seconds. Here, the liquid is subject to a centrifugal force, at 1800 rpm, of 109 RCF. Applied to milk, the time will be 3-5 seconds which will not have a major impact on a bio-assay. Analysis of viscous oils or honey or similar reagents would clearly need to be accounted for in microfluidics design. But within the range of normal reagents, viscosity can be disregarded as a properly which will effect system performance.*

*The only property which will have a major impact on the valves shown in this work, outside of contact angle for siphon valves, is the ability of the liquid to dissolve the water-dissolvable DFs used in this study. For example, in previous studies [1], [3], [4] we have had to tailor DFs to dissolve in EtOH / water solutions and have also leveraged that certain liquids (oils, fluorocarbons) do not dissolve polyvinyl alcohol films.*

*We have added the following text to Section 3:*

3. The device optimization is also not clear in the manuscript. How did the author choose the different frequencies for the operation?

*The siphon HLH valve (Figure 1) was designed through reviewing similar valves in literature and approximating their geometry. This architecture worked on the first attempt.*

*The DF derived HLH valve was a results of a number of iterations. The designs were primarily based on equations which have been previously derived for DF valves and for centrifuge-pneumatic siphon valves. In addition, we identified the DF derived PA valves (both HLH and LHL) as a key platform technology for our research group. We currently have two follow-on manuscripts in preparation which use different aspects of the LHL valves (previously published in Mishra et al.) and we hope many more will follow.*

*We provide the following comments to address the reviewers suggestion:*

*Section 3.1*

*Section 3.2*

*Section 4.2*

4. The efficacy of the device also lacks characterizations such as the filtration rate for the sample.
*In this work, we did not filter the sample as the calibrant is fully suspended in the reagent. However, we expect the reviewer is referring to the time taken to process the blood sample (separate out plasma). On our disc this took ~5 minutes and this is aligned with previously reported results (Nwankire et al.). As we are not reporting blood-based experiments, we have added the following comment to Section 4.2.1*

5. Some supplementary materials were not included in the submission.

*We note Reviewer #1 made a comment which was addressed in our ESI file. We believe it may have been corrupted. We will ensure it is attached to the primary document for consideration in this resubmission.*

**Additional Editor Comments:**

As an invited Academic Editor of PLOS ONE, I appreciate being assigned to this interesting manuscript. Please, consider each of the comments submitted by the Reviewers, taking special care of these two expanded here:

- Variations of media conditions (i.e., temperature, aging, irradiation, etc.), might alter the performance of the system? Either through the modification of the solving kinetics of the fluid, or through its interaction with the PMMA/PSA structure, its functionality may change. Indeed, although it is clear that extreme conditions may induce a complete degradability of the structure itself, could a mild variation also imperceptibly modify its surface, producing a bias? Perhaps a physicochemical analysis of the testing fluid, of the surface of the polymeric structure, and of the interaction between them might help.

*We thank the editor for this comment. As mentioned above, Kilcawley at al have hypothesised that the strobe light used in this investigation may have affected the results from blood samples. Conversely, Nwankire et al, who did not use a strobe light but used identical PSA, PMMA and testing conditions, observed excellent match between whole blood results and calibrant (processed on-disc, off-disc and in a hospital lab).*

*As mentioned, this work was conducted in 2014 and a large part of the team have moved onto other things. We believe the major innovation focus of this work is the high-low-high valves presented here. Investigation of the interaction of the media with the surfaces falls outside our resources / funding. We unfortunately cannot explore these interesting questions / suggestions as we might wish.*

- Why not testing the device with real blood or blood plasma examples? Furthermore, I would rather propose something more: could it be feasible to show a real plasma case where this device may succeed, in comparison to existing ones failing there?

*As described above, we did test with blood but unfortunately cannot publish our results in confidence as we do not have the complete data-sets. However, as discussed above, Nwankire et al has previously proven this assay functions well on the Lab-on-a-Disc.*

*While we appreciate our results in Figure 4 do not match perfectly between 100% calibrant (on-bench and on-chip), and so can be considered to be failing, we wish to re-iterate that the innovation focus of our work is on the valving technology rather than the assay itself. As shown in our supporting ESI videos, our overall disc architecture was capable of completing all the LUOs associated with this assay (high speed centrifugation for blood processing, medium speed for metering, mixing, and addition of 3 reagents at three different times) all in disc controlled entirely by the disc spindle motor (i.e. not requiring any ancillary equipment).*

*We hope that these novel valves will be of sufficient significance irrespective of the absence blood-based testing.*

**References**

[1]	N. A. Kilcawley *et al.*, “Dissolvable Film-Controlled Buoyancy Pumping and Aliquoting on a Lab-On-A-Disc,” *Processes*, vol. 11, no. 1, p. 128, 2023.

[2]	C. E. Nwankire *et al.*, “A portable centrifugal analyser for liver function screening,” *Biosens. Bioelectron.*, vol. 56, pp. 352–358, 2014.

[3]	R. Mishra *et al.*, “Plant pathogen detection on a lab-on-a-disc using solid-phase extraction and isothermal nucleic acid amplification enabled by digital pulse-actuated dissolvable film valves,” *Anal. Chim. Acta*, vol. 1258, p. 341070, 2023.

[4]	D. J. Kinahan *et al.*, “Centrifugally automated Solid-Phase Extraction of DNA by immiscible liquid valving and chemically powered centripetal pumping of peripherally stored reagents,” *Biosens. Bioelectron. X*, vol. 9, p. 100085, 2021.
